# Inherited predisposition to breast cancer in the Carolina Breast Cancer Study

**DOI:** 10.1038/s41523-020-00214-4

**Published:** 2021-01-21

**Authors:** Tom Walsh, Suleyman Gulsuner, Ming K. Lee, Melissa A. Troester, Andrew F. Olshan, H. Shelton Earp, Charles M. Perou, Mary-Claire King

**Affiliations:** 1 Department of Medicine and Department of Genome Sciences, University of Washington, Seattle, WA 98195 USA; 2 Lineberger Comprehensive Cancer Center, University of North Carolina, Chapel Hill, NC 27514 USA

**Keywords:** Cancer genetics, Cancer genetics

## Abstract

The Carolina Breast Cancer Study (CBCS) phases I–II was a case-control study of biological and social risk factors for invasive breast cancer that enrolled cases and controls between 1993 and 1999. Case selection was population-based and stratified by ancestry and age at diagnosis. Controls were matched to cases by age, self-identified race, and neighborhood of residence. Sequencing genomic DNA from 1370 cases and 1635 controls yielded odds ratios (with 95% confidence limits) for breast cancer of all subtypes of 26.7 (3.59, 189.1) for *BRCA1*, 8.8 (3.44, 22.48) for *BRCA2*, and 9.0 (2.06, 39.60) for *PALB2*; and for triple-negative breast cancer (TNBC) of 55.0 (7.01, 431.4) for *BRCA1*, 12.1 (4.18, 35.12) for *BRCA2*, and 10.8 (1.97, 59.11) for *PALB2*. Overall, 5.6% of patients carried a pathogenic variant in *BRCA1, BRCA2, PALB2*, or *TP53*, the four most highly penetrant breast cancer genes. Analysis of cases by tumor subtype revealed the expected association of TNBC versus other tumor subtypes with *BRCA1*, and suggested a significant association between TNBC versus other tumor subtypes with *BRCA2* or *PALB2* among African-American (AA) patients [2.95 (1.18, 7.37)], but not among European-American (EA) patients [0.62 (0.18, 2.09)]. AA patients with pathogenic variants in *BRCA2* or *PALB2* were 11 times more likely to be diagnosed with TNBC versus another tumor subtype than were EA patients with pathogenic variants in either of these genes (*P* = 0.001). If this pattern is confirmed in other comparisons of similarly ascertained AA and EA breast cancer patients, it could in part explain the higher prevalence of TNBC among AA breast cancer patients.

## Introduction

The Carolina Breast Cancer Study (CBCS) was designed in the early 1990s to evaluate genetic and social risk factors for invasive breast cancer^1^. Among the important results of the CBCS was elucidation of the relationship among breast tumor subtype, race, and survival^2^. The CBCS was also the setting of the first survey of *BRCA1* mutation frequency in any population, based on the first CBCS participants^3^. However, until now, the CBCS cohort was never comprehensively sequenced for mutations in genes predisposing to breast cancer.

The CBCS cohort is uniquely valuable in several ways for evaluation of genetic influences on breast cancer risk. First, enrollment for CBCS phases I–II occurred before genetic screening was available to this population, so women with mutations were not protected from becoming cases by prior genetic testing and risk-reducing surgery (although even now only a small fraction of *BRCA1* or *BRCA2* mutation carriers are identified prior to diagnosis). Second, sampling of cases was stratified so that numbers of European-American (EA) cases and African-American (AA) cases were approximately equal, and numbers of cases diagnosed before and after age 50 years were approximately equal^4^. Third, controls were ascertained specifically for this project and were carefully matched to cases by ancestry, age, and neighborhood of residence. Fourth, DNA samples were collected at enrollment, within 6 months of diagnosis, so participation was minimally subject to survival bias, and DNA was still available from the vast majority of participants.

We undertook to sequence all known and candidate breast cancer genes in all cases and controls from CBCS phases I–II. Our goals were to estimate the risks of invasive breast cancer and of triple-negative breast cancer (TNBC) associated with pathogenic variants in these genes and to evaluate the relationships among inherited predisposition, ancestry, and tumor subtype.

## Results

### Features of the cohort

Demographic and clinical features of the 1370 cases and 1635 controls are indicated in Table 1. As reported previously from the CBCS^2^, TNBC was more frequent among AA cases (178 of 509, 35%) than among EA cases (127 of 684, 19%); OR = 2.35, 95% CI (1.80, 3.06). Similarly, Basal-like and marker-negative tumor subtypes, which are associated with TNBC, were more frequent among AA cases than among EA cases: OR = 1.92 (1.35, 2.73) for Basal-like tumors and OR = 1.83 (1.20, 2.77) for tumors negative for all five markers. Reciprocally, Luminal A and Luminal B subtypes were more frequent among EA cases than among AA cases (OR = 1.66 [1.27, 2.18] for Luminal A and OR = 1.80 [1.11, 2.90] for Luminal B). Tumor grade at diagnosis was significantly poorer for AA cases than for EA cases (*P* = 0.002).

**Table 1:** Features of CBCS breast cancer cases and controls^a^.

	Cases	Controls
Total	1370 (1.000)	1635 (1.000)
Age at dx or enrollment		
20–29	23 (0.017)	10 (0.006)
30–39	212 (0.155)	190 (0.116)
40–49	500 (0.365)	577 (0.353)
50–59	269 (0.196)	385 (0.235)
60–69	256 (0.187)	321 (0.196)
70–74	110 (0.080)	152 (0.093)
Ancestry by self-report		
African-American (AA)	586 (0.428)	592 (0.362)
European-American (EA)	765 (0.558)	1016 (0.621)
Other	19 (0.014)	27 (0.017)
Menopausal status		
Pre-menopausal	606 (0.528)	624 (0.465)
Post-menopausal	542 (0.472)	719 (0.535)
Did not specify	221 (–)	292 (–)
1^o^ family history		
Breast and ovarian cancer	8 (0.006)	4 (0.003)
Breast cancer only	213 (0.160)	194 (0.122)
Ovarian cancer only	26 (0.020)	29 (0.018)
Neither	1083 (0.814)	1365 (0.857)
Unknown	38 (–)	43 (–)
TNBC		
Yes	307 (0.254)	
No	904 (0.746)	
Unknown	159 (–)	
Tumor subtype		
Basal-like	156 (0.179)	
Luminal A	475 (0.545)	
Luminal B	84 (0.096)	
HER2 + ER−	51 (0.059)	
Negative for all markers	105 (0.121)	
Unknown	499 (–)	
Grade		
Grade 1	152 (0.280)	
Grade 2	165 (0.304)	
Grade 3	225 (0.415)	
Unknown	809 (–)	
Nodal involvement		
Yes	466 (0.354)	
No	849 (0.646)	
Unknown	36 (–)	

^a^In parentheses, proportions of participants with information.

### Genetics

For case-control comparisons, odds ratios (with 95% confidence intervals) for breast cancer of all subtypes combined were 26.7 (3.59, 189.1) for *BRCA1*; 8.8 (3.44, 22.48) for *BRCA2*; and 9.0 (2.06, 39.60) for *PALB2*. Case-control odds ratios for TNBC were 55.0 (7.01, 431.4) for *BRCA1*; 12.1 (4.18, 35.12) for *BRCA2*; and 10.8 (1.97, 59.11) for *PALB2* (Table 2). Overall, 5.6% of patients, including 7.6% (55 of 721) patients diagnosed before age 50 and 3.4% (22 of 649) patients diagnosed between ages 50 and 74, carried a pathogenic variant in *BRCA1, BRCA2, PALB2*, or *TP53*, the four most highly penetrant breast cancer genes. Odds ratios for other genes had very wide confidence limits (Supplementary Table 1). Odds ratios for breast cancer and *BRCA1*, both for all subtypes and for TNBC, were higher than generally cited for American women (e.g., ref. ^5^). Odds ratios for breast cancer and *PALB2* were similar to odds ratios for breast cancer and *BRCA2*, and higher than odds ratios previously reported for *PALB2*^6^. Analysis of *BRCA2* and *PALB2* in the CBCS included only mutations leading to complete loss- of-function, whereas previous analysis of *PALB2* included both truncating mutations and in-frame deletions, which have a more modest effect on risk^7^.

**Table 2:** Odds ratios for pathogenic variants in high-penetrance breast cancer genes for breast cancer of all subtypes and for TNBC compared to controls.

Gene	All cases OR^a^ (95% CI)	TNBC OR^a^ (95% CI)	All cases^b^	TNBC^b^	Controls^b^
BRCA1	26.67 (3.59, 189.1)	55.02 (7.01, 431.4)	22 (0.016)	10 (0.033)	1 (0.001)
BRCA2	8.80 (3.44, 22.48)	12.11 (4.18, 35.12)	36 (0.026)	11 (0.036)	5 (0.003)
PALB2	9.04 (2.06, 39.60)	10.78 (1.97, 59.11)	15 (0.011)	4 (0.013)	2 (0.001)
TP53	*P* = 0.015	*P* = 0.11	5 (0.004)	2 (0.007)	0 (0.000)
Any of above			78 (0.057)	27 (0.088)	8 (0.005)
Total			1370 (1.000)	307 (1.000)	1635 (1.000)

^a^Odds ratios (OR) and confidence intervals (CI) adjusted for age and ancestry.

^b^Numbers and proportions of all cases, TNBC cases, and controls with mutations in each gene.

**Fig. 1:**
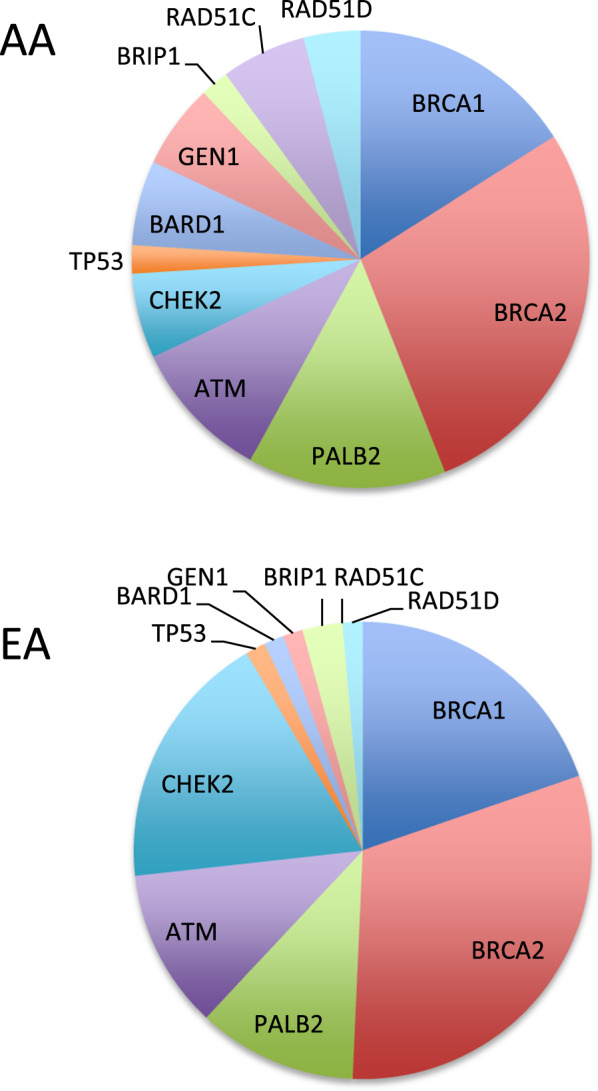
Genes responsible for inherited breast cancer in the Carolina Breast Cancer Study. The upper panel indicates genes with pathogenic or likely pathogenic mutations in African-American (AA) patients. The lower panel indicates genes with pathogenic or likely pathogenic mutations in European-American (EA) patients.

Both locus and allelic heterogeneity were high, with 150 different loss-of-function mutations in 24 different genes (Supplementary Tables 2 and 3). (Of the 25 genes included in the analysis, only *PTEN* had no case or control with a loss-of-function mutation.) Of the 150 different pathogenic or likely pathogenic variants, 16% (24 of 150) appeared in more than one participant; all others were singletons. On the other hand, with the exception of the European founder allele *CHEK2 c.1229delC (1100delC)*, distributions of breast cancer genes were similar for AA and EA cases (Fig. 1).

Pathogenic and likely pathogenic variants in *TP53* were present in five cases and no controls. Patients with TP53 were diagnosed with breast cancer at ages 23, 26, 26, 30, and 31 years. No information was available on other Li-Fraumeni syndrome cancers in these families, nor was DNA available from parents to determine if any of these variants were *de novo*. The association of *BARD1* with TNBC was marginally significant (Table 2), even though the number of *BARD1* mutation carriers was very small (Supplementary Tables 1, 2, 3). Several studies, both epidemiological and experimental, suggest that *BARD1* may be a TNBC gene^8,9^, and the CBCS results support that observation. Risk associated with *ATM* was within the range previously reported^10^, but not statistically significant. No significant increase in risk was observed for carriers of mutations in *CHEK2*, even if only truncating mutations were included in analysis, not because such mutations were rare, but because both cases and controls carried truncating mutations in *CHEK2* (Supplementary Tables 2 and 3). No TNBC patients carried a *CHEK2* truncating mutation, consistent with previous reports^9^. For the other 18 genes, too few participants carried pathogenic or likely pathogenic variants to make gene-specific inferences about risk.

### Tumor subtype and patient genotype

Information was available for the five tumor markers described above for tumors of 871 of the 1370 cases (Table 3, Supplementary Table 4). Of the 871 tested tumors, 18% (156) were Basal-like, 55% (475) were Luminal A, 10% (84) were Luminal B, 6% (51) were HER + ER−, and 12% (105) were negative for all five markers. Results from CBCS recapitulated with marginal significance the established relationship between tumor subtype and patient genotype^11^: that tumors of patients with *BRCA1* mutations are more likely to be Basel-like (6 of 16, 37.5%) than were tumors from patients of other genotypes (150 of 855, 17.5%; OR = 2.82, [1.01, 7.88]). Given the limited numbers of tumors with defined subtypes among patients with germline mutations, no other associations with host genotype were significant. It would be informative to extend germline genomic analysis to the larger series of CBCS patients whose tumors have been evaluated with current approaches to expression profiling^12–14^.

**Table 3:** Tumor subtypes of CBCS patients^a^.

Gene	Basal-like	Luminal A	Luminal B	HER2 + ER−	All negative	Total
BRCA1	6 (0.038)	5 (0.011)	2 (0.024)	0 (0.000)	3 (0.029)	16 (0.018)
BRCA2	5 (0.032)	12 (0.025)	4 (0.048)	0 (0.000)	5 (0.048)	26 (0.030)
PALB2	3 (0.019)	5 (0.011)	1 (0.012)	0 (0.000)	0 (0.000)	9 (0.010)
CHEK2	0 (0.000)	9 (0.019)	2 (0.024)	0 (0.000)	0 (0.000)	11 (0.013)
ATM	2 (0.013)	2 (0.004)	2 (0.024)	0 (0.000)	1 (0.010)	7 (0.008)
Other gene	5 (0.032)	21 (0.044)	2 (0.024)	2 (0.039)	3 (0.029)	33 (0.038)
No mutation	136 (0.872)	425 (0.895)	71 (0.845)	49 (0.961)	94 (0.895)	775 (0.890)
Total	156 (1.000)	475 (1.000)	84 (1.000)	51 (1.000)	105 (1.000)	871 (1.000)

^a^In parentheses, proportions of tumors of each subtype with mutation.

### TNBC, patient genotype, and ancestry

As expected, a diagnosis of TNBC versus any other tumor subtype was more frequent among patients with pathogenic variants in *BRCA1* than among patients with no pathogenic variant in *BRCA1, BRCA2*, or *PALB2*, both for AA patients and for EA patients (Table 4), although given small numbers, the association for all patients was significant only by log-linear analysis, adjusting for ancestry and age.

**Table 4:** Odds ratios for TNBC versus breast cancer of other subtypes for patients with pathogenic variants in BRCA1, BRCA2, or PALB2^a^.

Gene	TNBC	Not TNBC	OR (95% CI)	*P*-value
African-American patients				
BRCA1	4 (0.022)	2 (0.006)	3.78 (0.69, 20.9)	0.19^b^
BRCA2	8 (0.045)	5 (0.015)	3.15 (1.01, 9.79)	0.04
PALB2	4 (0.022)	3 (0.009)	2.63 (0.58, 11.9)	0.24
BRCA2 or PALB2	12 (0.067)	8 (0.024)	2.95 (1.18, 7.37)	0.018
Total	178 (1.000)	331 (1.000)		
European-American patients				
BRCA1	7 (0.055)	6 (0.012)	5.36 (1.77, 16.2)	0.004^b^
BRCA2	3 (0.024)	15 (0.027)	0.90 (0.26, 3.17)	ns
PALB2	0	7 (0.013)	0	ns
BRCA2 or PALB2	3 (0.024)	22 (0.039)	0.62 (0.18, 2.09)	ns
Total	127 (1.000)	557 (1.000)		

*OR* odds ratio, *CI* confidence interval, *P P*-value.

^a^TNBC status could be assigned for 86% (509/591) of tumors of African-American patients and 90% (684/760) of tumors of European-American patients.

^b^Log-linear analysis for all patients of BRCA1 genotype × TNBC status × ancestry yields *P* = 0.0045 for the BRCA1-TNBC association, controlling for ancestry.

In contrast, the relationship between a diagnosis of TNBC versus any other tumor subtype and *BRCA2* and *PALB2* might be different for AA and EA patients (Table 4). TNBC versus other tumor subtype was significantly more frequent among AA patients with pathogenic variants in *BRCA2* or *PALB2* than among AA cases with no such variants (OR = 2.95 [1.18, 7.37]). In contrast, TNBC versus other tumor subtype was not more frequent among EA breast cancer cases with pathogenic variants in *BRCA2* or *PALB2* than among EA cases with no such variants (OR = 0.62 [0.18, 2.09]). Of patients with pathogenic variants in *BRCA2* or *PALB2*, 60% of AA patients (12/20) versus 12% of EA patients (3/25) presented with TNBC versus any other tumor subtype (*P* = 0.001).

## Discussion

The CBCS was a comprehensive population-based case-control study of risk factors for breast cancer, with stratified sampling to yield approximately equal numbers of AA and EA cases and of cases diagnosed before versus after age 50; and with rigorous matching of controls to cases by age, ancestry, and neighborhood of residence. Enrollment of CBCS phases I–II occurred in the 1990s, before genetic testing was generally available, so case ascertainment was not subject to selection bias due to removal of the highest risk women from the case pool by virtue of risk-reducing interventions. The >20 years since enrollment also led to the principal limitations of the study: information on tumor hormone receptor status was incomplete, known for 87% (1193 of 1370) patients. Receptor status was evaluated centrally, and was recorded only as positive or negative, so whether the number of borderline ER-positive cases differed for AA versus EA patients is not known. Information on tumor subtype was even more incomplete, known for 64% (871 of 1370) patients. However, thanks to collection of DNA at the time of enrollment, within 6 months of diagnosis, genetic analyses could be undertaken with minimal survival bias. The CBCS cohort offered a unique opportunity to evaluate inherited predisposition to breast cancer using modern genomics tools.

Sequencing all known and multiple candidate breast cancer genes for CBCS cases and controls revealed three observations of general interest in cancer genetics. First, the same genes were involved, and (with the exception of *CHEK2*) in similar proportions, in inherited predisposition to breast cancer in AA and EA women. This result was to be expected but had not previously been shown in a population-based cohort including high proportions of both EA and AA patients.

Second, among all women in the cohort, 5.6% of patients carried a pathogenic variant in *BRCA1, BRCA2, PALB2*, or *TP53*. The high prevalence of inherited disease due to these high-penetrance genes makes a compelling argument for genetic testing for all breast cancer patients diagnosed before age 75 (the upper age threshold for participation in the CBCS). Testing for somatic mutations in tumors can reveal purely somatic loss-of-function of the same genes. In tumors evaluated for The Cancer Genome Atlas (TGCA), including tumors from participants in later phases of the CBCS, ~20% of basal-like tumors included a predisposing mutation in *BRCA1* or *BRCA2*, either germline or somatic^15^, suggesting that these patients could benefit from genotype-targeted therapy.

Third, TNBC rather than some other tumor subtype as a patient’s first breast cancer diagnosis may be more strongly associated with *BRCA2* and *PALB2* among AA patients than among EA patients. The association of TNBC and patient genotype among AA patients in the CBCS recapitulated the pattern that we observed in two other studies, one of Nigerian patients and one of African-American patients^16,17^. Considering patients diagnosed before age 50 (as were most Nigerian patients), among Nigerian patients with pathogenic variants in *BRCA1*, 67% were diagnosed with TNBC^16^, compared to 60% of CBCS AA patients and 55% of CBCS EA patients, whereas among Nigerian patients with pathogenic variants in *BRCA2* or *PALB2*, 50% were diagnosed with TNBC^16^, compared to 62% of CBCS AA patients and 14% of CBCS EA patients. In another, small series, among 180 AA patients seen at the University of Chicago cancer genetics clinic for suspicion of inherited predisposition to cancer, but regardless of tumor hormone status, odds ratio for association of TNBC rather than another tumor subtype with *BRCA2 or PALB2* was 4.92 (1.18, 20.46)^17^. For both these cohorts, the numbers of patients with tumors of known hormone receptor status and with pathogenic variants in *BRCA2* or *PALB2* were small. It will be important to test this association in other series of patients of African-American and African ancestry.

The mechanism for a stronger association of a diagnosis of TNBC versus other tumor subtypes with *BRCA2* and *PALB2* among patients of African and AA ancestry is not obvious. It is unlikely to reflect differences in the mutant alleles themselves, because in all populations, all alleles included in the analyses led to stops and were distributed across the length of both genes. Nor did we detect any inherited alleles modifying the association of TNBC with mutation in *BRCA2* or *PALB2*, although modifying alleles could certainly exist in genes other than those sequenced. Another possibility is that somatic events, either genomic mutations or methylation of tumors, could be more severe among AA and Nigerian patients. Furthermore, if all breast tumors are hormone receptor positive before developing as TNBC, then the stronger association might be explained by diagnosis of breast cancers later in tumor development for AA and Nigerian patients compared to EA patients. This diagnostic differential could be due to more rapid progression of tumors among *BRCA2* or *PALB2* mutation carriers of African ancestry, or to differences in access to diagnosis, or both. Alternatively, it is possible that *BRCA2* and *PALB2* are biologically associated with TNBC as the first diagnosis for both AA and EA patients, but that, for unknown reasons, EA patients in the CBCS had exceptionally low prevalence of TNBC. For example, in a recent large series of breast cancer patients undergoing genetic testing by Ambry Genetics^18^, 66% of EA patients with pathogenic variants in *BRCA1* reported a diagnosis of TNBC, and 30% of EA patients with pathogenic variants in *BRCA2* or *PALB2* reported a diagnosis of TNBC, both higher frequencies of TNBC than among EA patients in the CBCS. Comparable data for TNBC among AA patients in the Ambry cohort were not reported.

In summary, case-control comparisons yielded high estimates of relative risk for breast cancer, and even higher estimates for TNBC, for *BRCA1*, *BRCA2*, and *PALB2* among both EA and AA women. Among cases, a first diagnosis of TNBC rather than any other breast cancer subtype was associated with *BRCA1* for both AA and EA patients. In contrast, a first diagnosis of TNBC rather than any other breast cancer subtype was associated with *BRCA2* or *PALB2* for AA patients, but not for EA patients. We look forward to analyses of TNBC and inherited genotype among other series of AA patients and EA patients who were ascertained in the same way. If the same pattern is observed, it could contribute to understanding the higher prevalence of TNBC among AA women with breast cancer.

## Methods

### Subjects

Between 1993 and 1999, CBCS phases I–II enrolled women diagnosed with invasive breast cancer between ages 20 and 74, identified by the North Carolina Central Cancer Registry as residents of one of the 24 counties of central North Carolina^1^. Case sampling was stratified to yield approximately equal numbers of AA and EA cases, and approximately equal numbers of patients with diagnoses before and after age 50 years. Controls were ascertained through public databases and matched to cases for age within 5 years, self-identified race, and neighborhood of residence. From all participants, CBCS collected information on multiple reproductive and physical risk factors. Family history of cancer was recorded but was not a criterion for inclusion in the study. Participants also provided samples of peripheral whole blood from which DNA was extracted for subsequent genetic analyses^19^. This project was approved by the institutional review boards of the Lineberger Cancer Center at University of North Carolina and of the University of Washington. Participants in the CBCS signed written informed consent at enrollment, including for use of their DNA.

From CBCS phases I–II, 3104 DNA samples were available, including 1412 samples from cases and 1692 samples from controls. Quality control tests revealed degraded DNA from 96 samples (40 from cases and 56 from controls); these samples were omitted. Subsequent genomic analysis revealed two case samples and one control sample to be intentional duplicates of other samples. Genomic analysis was therefore carried out for 1370 different cases and 1635 different controls. Before sequencing, each sample was provided a sequential number, and all sequencing, alignment, and variant interpretation were undertaken without knowledge of case-control status.

### Tumor analysis

Tumors of patients in CBCS phases I–II were characterized for five markers: ER, PR, HER2 (by immunohistochemistry), CK5/6, and HER1/DEF1. Tumor markers were evaluated for a previous study, in which details of sample ascertainment and storage, antibodies, and immunochemistry were described^2^. Tumor subtypes were classified as Basal-like (ER negative and PR negative and HER2 negative and either HER1 positive or CK5/6 positive), Luminal A (HER2 negative and either ER positive or PR positive), Luminal B (HER2 positive and either ER positive or PR positive), HER + ER− (HER2 positive and ER negative and PR negative); or negative for all five markers.

### Genomics

DNA extracted from peripheral blood samples of cases and controls was sequenced using BROCA, a next-generation capture and sequencing protocol that includes all known genes harboring mutations predisposing to breast cancer^20^. BROCA sequencing was carried out for 96 samples per lane, yielding >300-fold depth of coverage for each sample. For each gene on the panel, BROCA targets coding sequences, introns (minus Alu repeats), UTRs, and flanking intergenic regions. The classes of mutations identified are single nucleotide variants (SNVs) and small indels, whether in exons, regulatory regions, or introns; exonic deletions and duplications, and large rearrangements (copy number variants, or CNVs)^21^. BROCA has been used to evaluate inherited predisposition to breast, ovarian, prostate, and related cancers^17,22–27^.

A total of 25 genes were evaluated: *BRCA1*, *BRCA2*, *PALB2*, *TP53*, *PTEN*, *CDH1*, *ATM*, *BARD1*, *BRIP1*, *CHEK2*, *GEN1*, *RAD51C*, *RAD51D*, *ATR*, *CHEK1*, *CTNNA1*, *FAM175A*, *FANCM*, *MRE11A*, *NBN*, *RAD51B*, *RECQL*, *RINT1*, *SLX4*, and *XRCC2*. The strength of evidence for causal association with breast cancer obviously varies a great deal from gene to gene, as do the empiric risks to carriers of pathogenic variants. Analyses included only pathogenic and likely pathogenic mutations in each gene; that is, frameshifts, nonsense mutations (except at the extreme 3′ end of some genes), genomic deletions leading to a stop, splice and enhancer mutations shown experimentally to lead to a stop, and missense mutations shown experimentally to lead to loss of a critical function of the protein. Our protocols for sequencing, alignment, and variant interpretation using BROCA have been described^22^. In addition, 128 ancestry-informative markers^28^ were included in the BROCA panel and each participant was assigned to the ancestral group with which her sample was most closely aligned^29^. For purposes of this project, ancestral groups were African-American, European-American, Asian-American, and Native American. For nearly all participants, ancestral groups defined by ancestry-informative markers corresponded to ancestry identified by self-report.

### Statistics

Frequencies of pathogenic and likely pathogenic variants among cases and among controls, and among cases with tumors of different subtypes, were compared by log-linear analyses to adjust for different numbers of controls per case from different ancestral groups and different age strata. Log-linear analyses were also used to test for interactions between genotype, case-control status, and race. Odds ratios (OR), 95% confidence intervals (CI), and *P*-values were calculated from regression analyses or from Fisher exact tests for rare events, as appropriate. All tests were two-tailed.

### Reporting summary

Further information on experimental design is available in the Nature Research Reporting Summary linked to this paper.

## Supplementary information

Supplemental tables

Reporting Summary Checklist

## Data Availability

Pathogenic variants reported for the first time in this study are available in the NCBI ClinVar repository: https://identifiers.org/clinvar.submission:SCV001443125, https://identifiers.org/clinvar.submission:SCV001443126, https://identifiers.org/clinvar.submission:SCV001443217, https://identifiers.org/clinvar.submission:SCV001443129. CBCS demographic and clinical data are not publicly available in order to protect patient privacy, but within IRB constraints will be made available on reasonable request from the corresponding author (mcking@uw.edu). Other data supporting the findings of this study are included in the supplementary tables of the article. The data generated and analyzed during this study are described in figshare: 10.6084/m9.figshare.13347380.
