# Supplementary material for: Inherited predisposition to breast cancer in the Carolina Breast Cancer Study
Source: NPJ Breast Cancer. 2021 Jan 21;7:6. doi: 10.1038/s41523-020-00214-4 (PMC7820260; doi:10.1038/s41523-020-00214-4)
Supplement: Supplementary file 1 — Supplemental tables [file 41523_2020_214_MOESM1_ESM.pdf]

Inherited Predisposition to Breast Cancer in the Carolina Breast Cancer Study

Supplementary Tables

Tom Walsh, PhD; Suleyman Gulsuner, MD, PhD; Ming K. Lee, PhD;

Melissa A. Troester, PhD; Andrew F. Olshan, PhD; H. Shelton Earp, MD;

Charles M. Perou, PhD; Mary-Claire King, PhD

Table S1. Odds ratios for pathogenic variants in moderate and lower penetrance genes for breast cancer of all subtypes and for TNBC compared to controls

	Breast cancer all subtypes		TNBC		Numbers and proportions of participants					
Gene	OR*	(95% C.I.)	OR*	(95% C.I.)	All cases (N=1370)		TNBC (N=307)		Controls (N=1635)	
ATM	2.10	(0.88, 5.02)	2.01	(0.53, 7.61)	13	0.010	3	0.010	8	0.005
CHEK2^	1.41	(0.63, 3.17)	-		13	0.010	0	0.000	11	0.007
BARD1	4.79	(0.53, 42.86)	10.71	(0.97, 118.5)	4	0.003	2	0.007	1	0.001
BRIP1	1.19	(0.24, 5.92)	1.78	(0.18, 17.15)	3	0.002	1	0.003	3	0.002
GEN1	P = 0.029		-		4	0.003	0	0.000	0	0.000
RAD51C	3.59	(0.37, 34.51)	5.34	(0.33, 85.61)	3	0.002	1	0.003	1	0.001
RAD51D	1.79	(0.30, 10.74)	2.67	(0.24, 29.52)	3	0.002	1	0.003	2	0.001

**Odds ratios adjusted for age and ancestry
^Truncating mutations only

Table S2. Pathogenic and likely pathogenic variants in CBCS cases (N = 1370) and controls (N = 1635)

Gene	Number of different mutations	Cases with mutation	Controls with mutation
BRCA1	19	22	1
BRCA2	33	36	5
PALB2	11	15	2
TP53	4	5	0
ATM	16	13	8
ATR	2	0	3
BARD1	3	4	1
BRIP1	4	3	3
CDH1	1	0	1
CHEK1	1	0	1
CHEK2 stops	4	13	11
CHEK2 p.I157T	1	0	3
CTNNA1	3	3	2
FAM175A	4	0	4
FANCM	6	9	8
GEN1	4	4	0
MRE11A	6	1	5
NBN	2	1	1
RAD51B	3	1	2
RAD51C	4	3	1
RAD51D	4	3	2
RECQL	4	3	2
RINT1	6	2	4
SLX4	3	0	3
XRCC2	2	1	1

Table S3. P and LP variants in all genes sequenced on BROCA in CBCS cases and controls

chr	start (hg19)	end (hg19)	Gene	cDNA	protein	Number of cases	Number controls	Cases' ages at diagnosis	Controls' ages at enrollment
2	17,954,052		GEN1	c.953(+1)G>A	287X	1		51	
2	17,954,567		GEN1	c.1071(+1)G>T		1		59	
2	17,955,645		GEN1	c.1179delCT	393fs	1		32	
2	17,962,508		GEN1	c.2029 C>T	R677X	1		48	
2	215,595,135		BARD1	c.2001G>C	568X	1		47	
2	215,645,382		BARD1	c.1216C>T	R406X	1	1	51	35
2	215,645,386		BARD1	c.1212C>G	Y404X	2		24, 59	
3	142,215,250		ATR	c.5851C>T	R1951X		2	50, 51	
3	142,231,101		ATR	c.4852(+1)G>A	1559X		1		62
4	84,383,708		FAM175A	c.1143insA	A382fs		1		62
4	84,383,745		FAM175A	c.1106insG	R369fs		1		63
4	84,383,756		FAM175A	c.1096 C>T	Q366X		1		43
4	84,383,841		FAM175A	c.1011delA	P337fs		1		44
5	138,117,698		CTNNA1	c.85delT	L29fs	2	1	48, 68	73
5	138,117,719		CTNNA1	c.105(+1)G>C			1		51
5	138,145,894		CTNNA1	c.468(+1)G>T		1		36	
7	105,171,883	152,373,689	RINT1/XRCC2	del two complete genes			1		40
7	105,182,891		RINT1	c.310C>T	R104X		1		38
7	105,187,693		RINT1	c.752insTGTT	T251fs		1		42
7	105,187,739		RINT1	c.798C>G	Y266X	1		40	
7	105,204,391		RINT1	c.1883delA	E628fs		1		49
7	105,205,851		RINT1	c.2014del13	672fs	1		45	
7	152,345,090	152,373,689	XRCC2	del complete gene		1		46	
8	90,955,548		NBN	c.2117C>G	S706X		1		54
8	90,983,442		NBN	c.657delTTTGT	219fs	1		61	
11	94,167,605	94,171,026	MRE11A	del exons 17+18			1		35
11	94,180,397		MRE11A	c.1771C>T	Q591X		1		44
11	94,180,454		MRE11A	c.1636C>T	R546X		1		67
11	94,180,454		MRE11A	c.1714C>T	R572X		1		38
11	94,200,987		MRE11A	c.1090C>T	R364X	1		42	
11	94,204,762		MRE11A	c.822delAA	274fs		1		46
11	108,098,600		ATM	c.170G>A	W57X	1	1	46	46
11	108,106,564		ATM	c.496(+3)A>G	del 55 aa		1		45
11	108,121,480		ATM	c.1288delTG	430fs	1		49	
11	108,121,531		ATM	c.1339C>T	R447X	1	1	74	55
11	108,121,561		ATM	c.1369C>T	R457X	1		32	
11	108,121,594		ATM	c.1402delAA	468fs	1	1	41	70
11	108,121,753		ATM	c.1561delAG		1		24	
11	108,137,979		ATM	c.2548G>T	E850X	1		46	
11	108,139,191		ATM	c.2693T>G	L898X	1		60	
11	108,175,528		ATM	c.5623C>T	R1875X	1		38	
11	108,183,136		ATM	c.5919(-2) A>G			1		50
11	108,190,736		ATM	c.6403insTT	L2135fs	1		43	
11	108,202,604		ATM	c.7630(-2)A>C	del aa 2544-2596		1		39
11	108,203,613		ATM	c.7913G>A	W2638X	1	2	51	54, 63
11	108,203,621		ATM	c.7921C>T	Q2641X	1		58	
11	108,233,681	108,240,500	ATM	del exons 62-63		1		59	
11	125,497,593		CHEK1	c.157delAGAA	53fs		1		43
12	21,623,219		RECQL	c.1859C>G	S620X	1		56	56
12	21,624,539		RECQL	c.1489dupA	I497fs		1		73
12	21,627,774		RECQL	c.1355(+1)G>A			1		52
12	21,627,911		RECQL	c.1219C>T	R407X	2		35, 52	
13	32,900,691		BRCA2	c.572delAT	191fs	1		43	
13	32,905,149		BRCA2	c.775delAG	260fs	1		36	
13	32,907,071		BRCA2	c.1456C>T	Q486X	1		59	
13	32,907,269		BRCA2	c.1654delT	S552fs	3		44, 44, 58	
13	32,907,383		BRCA2	c.1768delTTTA	590fs	1		45	
13	32,907,447		BRCA2	c.1832C>A	S611X	1		32	
13	32,907,449		BRCA2	c.1834G>T	E612X	1		72	
13	32,911,150		BRCA2	c.2658delTG	886fs	1		56	
13	32,911,298		BRCA2	c.2806delAAAC	K936fs	1		59	
13	32,911,499		BRCA2	c.3007delCA	1003fs	2		64, 65	
13	32,911,899		BRCA2	c.3408ins279	ins Alu (279 bp)	1		38	
13	32,912,172		BRCA2	c.3680delTG	1227fs		1		68
13	32,912,338		BRCA2	c.3846delTG	1282fs	1	1	62	68
13	32,912,363		BRCA2	c.3871C>T	Q1291X	1		65	
13	32,913,425		BRCA2	c.4933delAAAG	1645fs	1		45	
13	32,913,457		BRCA2	c.4965C>G	Y1655X	1		40	
13	32,913,558		BRCA2	c.5067insA	A1689fs		1		46
13	32,913,795		BRCA2	c.5303delTT	1768fs	1		51	
13	32,914,755		BRCA2	c.6263delC	T2088fs	2		44, 57	
13	32,914,954		BRCA2	c.6462delTC	2154fs	1		34	
13	32,920,978		BRCA2	c.6952 C>T	R2318X	1		41	
13	32,929,058		BRCA2	c.7068delTC	2356fs	1		46	
13	32,930,667		BRCA2	c.7539insA	A2513fs	1		39	
13	32,930,687		BRCA2	c.7558C>T	R2520X	4		25 - 48	

chr	start (hg19)	end (hg19)	Gene	cDNA	protein	Number of cases	Number controls	Cases' ages at diagnosis	Controls' ages at enrollment
13	32,932,067		BRCA2	c.7805(+1)G>A		1		31	
13	32,937,458		BRCA2	c.8119A>G	2702X	1		46	
13	32,944,570		BRCA2	c.8363G>A	W2788X	1		33	
13	32,944,571		BRCA2	c.8364G>A	W2788X	1		49	
13	32,954,208		BRCA2	c.9182T>A	L3061X		1		59
13	32,968,863		BRCA2	c.9294C>G	Y3098X	1		41	
13	32,968,900		BRCA2	c.9331 G>T	E3111X		1		47
13	32,972,316		BRCA2	c.9666delT	C3222fs	1		67	
13	32,972,590		BRCA2	c.9940delA	K3314fs	1		49	
14	45,624,626		FANCM	c.1360ins10	E454fs	1	1	52	54
14	45,645,431		FANCM	c.3474delTT	1158fs		1		70
14	45,645,480		FANCM	c.3523C>T	Q1175X	1		58	
14	45,658,326		FANCM	c.5101C>T	Q1701X	3		49, 51, 60	
14	45,667,877		FANCM	c.5747delAG	1916fs		1		44
14	45,667,921		FANCM	c.5791C>T	R1931X	4	5	32 - 66	32 - 71
14	68,290,282		RAD51B	c.22C>T	R8X		1		52
14	68,292,293		RAD51B	c.197C>T	del aa 67-105	1		68	
14	68,301,915		RAD51B	c.315(+2) T>A	del aa 67-105		1		43
16	3,633,462		SLX4	c.4789 G>T	E1597X		1		47
16	3,641,170		SLX4	c.2469G>A	W823X		1		69
16	3,644,477		SLX4	c.2137C>T	R713X		1		56
16	23,614,792		PALB2	c.3549C>G	Y1183X	1		39	
16	23,619,212		PALB2	c.3323delA	Y1108fs	2		39, 56	
16	23,632,683		PALB2	c.3113G>A	W1038X	3	1	49, 49, 53	44
16	23,641,063		PALB2	c.2411delAG	804fs	2		63, 67	
16	23,641,089		PALB2	c.2386G>T	G796X	1		63	
16	23,646,388		PALB2	c.1479delC	493fs		1		57
16	23,646,826		PALB2	c.1037del5	346fs	1		63	
16	23,647,379		PALB2	c.487delAC	163fs	1		37	
16	23,647,416		PALB2	c.451 C>T	Q151X	2		35, 37	
16	23,649,207		PALB2	c.172del4	58fs	1		46	
16	23,649,420		PALB2	c.79G>T	E27X	1		48	
16	68,857,427		CDH1	c.2062delTG	688fs		1		31
17	7,573,979		TP53	c.1048C>G	L350V	2		26, 31	
17	7,577,022		TP53	c.916C>T	R306X	1		30	
17	7,577,556		TP53	c.725G>T	C242F	1		23	
17	7,579,555		TP53	c.125delATTTGATG	D42fs	1		26	
17	33,426,507	33,429,871	RAD51D	del exons 9-10			1		43
17	33,434,036		RAD51D	c.451C>T	Q151X		1		48
17	33,434,403		RAD51D	c.326insC	P109fs	2		64	
17	33,445,519		RAD51D	c.261(+1)G>A	50X	1		48	
17	41,197,797		BRCA1	c.5490delA	A1830fs	1		34	
17	41,201,157		BRCA1	c.5387C>A	S1796X	1		32	
17	41,209,079		BRCA1	c.5266dupC	Q1756fs	1		41	
17	41,215,363		BRCA1	c.5177delGAAA	1726fs	1	1	69	53
17	41,215,391		BRCA1	c.5153(-1)G>C		1		69	
17	41,219,136	41,223,743	BRCA1	del exons 16-17		1		36	
17	41,223,186		BRCA1	c.4745delA	D1582fs	1		41	
17	41,230,485	41,235,875	BRCA1	dup exon 13		1		53	
17	41,234,420		BRCA1	c.4357(+1)G>A		1		31	
17	41,234,451		BRCA1	c.4327C>T	R1443X	2		33, 38	
17	41,234,567		BRCA1	c.4211delT	1404fs	1		32	
17	41,243,680		BRCA1	c.3868A>T	K1290X	1		32	
17	41,243,800		BRCA1	c.3748G>T	E1250X	3		32, 35, 46	
17	41,244,057		BRCA1	c.3481del11	1161fs	1		39	
17	41,244,291		BRCA1	c.3257T>G	L1086X	1		32	
17	41,245,091		BRCA1	c.2457delC	S819fs	1		26	
17	41,245,210		BRCA1	c.2338C>T	Q780X	1		53	
17	41,246,162		BRCA1	c.1386delG	462fs	1		32	
17	41,256,277		BRCA1	c.303T>G	Y101X	1		40	
17	56,772,331		RAD51C	c.185delAA	Q62fs	1		48	
17	56,779,227	56,781,041	RAD51C	del exon 4		1		48	
17	56,780,555		RAD51C	c.572(-2)A>G		1		44	
17	56,807,221	56,810,396	RAD51C	del exon 2			1		47
17	59,760,757		BRIP1	c.3650G>A	W1217X		1		53
17	59,793,412		BRIP1	c.2392C>T	R798X	2	1	39	45
17	59,858,254		BRIP1	c.1741C>T	R581X	1		46	
17	59,930,000	59,941,000	BRIP1	del exons 1-4 (approx breakpoints)			1		41
22	29,091,723	29,127,928	CHEK2	dup exons 3-15		1		44	
22	29,091,857		CHEK2	c.1229delC (1100delC)	T367fs	11	9	30 - 73	41 - 62
22	29,121,087		CHEK2	c.470T>C	I157T		3		46, 53, 63
22	29,130,427		CHEK2	c.283C>T	R95X		2		47, 65
22	29,130,431		CHEK2	c.279G>A	W93X	1		47	

Table S4. Tumor subtypes and patient genotypes

	Basal-like		Luminal A		Luminal B		HER2+ ER-		Negative all		Total tumors	
Gene	N	prop	N	prop	N	prop	N	prop	N	prop	N	prop
Total	156	0.18	475	0.55	84	0.10	51	0.06	105	0.12	871	1.00
BRCA1	6	0.38	5	0.31	2	0.13	0	0.00	3	0.19	16	1.00
BRCA2	5	0.19	12	0.46	4	0.15	0	0.00	5	0.20	26	1.00
PALB2	3	0.33	5	0.56	1	0.11	0	0.00	0	0.00	9	1.00
CHEK2 stops	0	0.00	9	0.82	2	0.18	0	0.00	0	0.00	11	1.00
ATM	2	0.29	2	0.29	2	0.29	0	0.00	1	0.14	7	1.00
FANCM	0	0.00	3	0.60	2	0.40	0	0.00	0	0.00	5	1.00
Other gene	5	0.18	18	0.64	0	0.00	2	0.07	3	0.11	28	1.00
No mutation	136	0.18	425	0.55	71	0.09	49	0.06	94	0.12	775	1.00
